# Population of the Javan Gibbon (*Hylobatesmoloch*) in the Dieng Mountains, Indonesia: An updated estimation from a new approach

**DOI:** 10.3897/BDJ.11.e100805

**Published:** 2023-07-04

**Authors:** Salmah Widyastuti, Dyah Perwitasari-Farajallah, Entang Iskandar, Lilik B Prasetyo, Arif Setiawan, Nur Aoliya, Susan M Cheyne

**Affiliations:** 1 Animal Biosciences Program, Department of Biology, Faculty of Mathematics and Natural Sciences, IPB University, Bogor, Indonesia Animal Biosciences Program, Department of Biology, Faculty of Mathematics and Natural Sciences, IPB University Bogor Indonesia; 2 Department of Biology, Faculty of Mathematics and Natural Sciences, IPB University, Bogor, Indonesia Department of Biology, Faculty of Mathematics and Natural Sciences, IPB University Bogor Indonesia; 3 Primate Research Centre, IPB University, Bogor, Indonesia Primate Research Centre, IPB University Bogor Indonesia; 4 Department of Forest Resources Conservation and Ecotourism, Faculty of Forestry and Environment, IPB University, Bogor, Indonesia Department of Forest Resources Conservation and Ecotourism, Faculty of Forestry and Environment, IPB University Bogor Indonesia; 5 SwaraOwa, Yogyakarta, Indonesia SwaraOwa Yogyakarta Indonesia; 6 Faculty of Humanities, Social Sciences and Law, Oxford Brookes University, Oxford, United Kingdom Faculty of Humanities, Social Sciences and Law, Oxford Brookes University Oxford United Kingdom

**Keywords:** ASCR, density, point count, population size, triangulation

## Abstract

The Javan gibbon (*Hylobatesmoloch*) is endemic to the island of Java and its distribution is restricted from the western tip of Java to the Dieng Mountains in Central Java. Unlike the other known habitats that hold a large population of Javan gibbons, the Dieng Mountains have not been protected and experience various threats. This study, which was conducted in 2018 and 2021, aimed to provide an update of the current density and population size of Javan gibbons in Dieng after the most recent study in 2010 and to investigate their relationships with habitat characteristics (vegetation and elevation). The triangulation method and a new acoustic spatial capture-recapture method were used to estimate group density. A new approach for extrapolation, based on the habitat suitability model, was also developed to calculate population size. The results show that the Javan gibbon population in the Dieng Mountains has most likely increased. The mean group density in each habitat type was high: 2.15 groups/km^2^ in the low suitable habitat and 5.55 groups/km^2^ in the high suitable habitat. The mean group size (3.95 groups/km^2^, n = 20) was higher than those reported in previous studies. The overall population size was estimated to be 1092 gibbons. This population increase might indicate the success of conservation efforts during the last decade. However, more effort should be made to ensure the long-term future of this threatened species. Although the density significantly differed between habitat suitability types, it was not influenced by the vegetation structure or elevation. A combination of multiple variables will probably have a greater effect on density variation.

## Introduction

Indonesia is home to nine species of small apes (Roos et al. 2014). As the most densely populated mainland in Indonesia, Java supports only one species of gibbon, the Javan gibbon (Nijman 2004). This silvery gibbon is endemic to Java, is in the International Union for Conservation of Nature (IUCN) Red List as endangered and has a restricted distribution in the western half of Java due to the drier climate in the eastern region of Java (Kappeler 1984, Nijman 2020). The Javan gibbon natural distribution is from the western tip of Java, Ujung Kulon National Park, to the Dieng Mountains, Central Java. The latest study revealed that the remaining population of this species is composed of 4000–4500 individuals (Nijman 2004). The three largest populations are in Halimun Salak National Park (850–1320 gibbons), Ujung Kulon National Park (560 gibbons) and the Dieng Mountains (500–881 gibbons) (Nijman 2004, Setiawan et al. 2012). In contrast to the other habitats, the Dieng Mountains is an area that holds the largest Javan gibbon population outside protected areas. However, it faces more threats due to fragmentations.

Many studies on Javan gibbon ecology have been conducted, but only a few included the Dieng Mountains. The faunal survey in 1994 first reported the Javan gibbon population in Dieng, which was estimated to range from 519 to 577 gibbons living in the mountains (Nijman and Van Balen 1998, Nijman 2004). The next population survey in 2003 estimated 595 gibbons in Dieng (Djanubudiman et al. 2004) and the latest, more extensive survey, in 2010, revealed a larger distribution and population of approximately 881 gibbons inhabiting four forest blocks in Dieng (Setiawan et al. 2012). After more than a decade, no study has evaluated the Javan gibbon population in Dieng. As forest extraction and anthropogenic activities continue in this unprotected landscape despite conservation efforts from several communities, the Javan gibbon population might have decreased, increased or remained stable. Therefore, the aim of this study was to estimate the density and population size of Javan gibbons in the Dieng Mountains to provide an updated assessment and baseline conservation data. In this study, the triangulation method, based on auditory data, was used to estimate the group density (Brockelman and Ali 1987), as it is believed to be the most efficient method for surveying gibbons in hilly terrains like the Dieng Mountains, to cover more area and to detect more gibbon groups than visual methods (Brockelman 2019). The standard triangulation (Brockelman and Ali 1987) and the newest acoustic spatial capture-recapture (ASCR) method (Stevenson et al. 2021) were generated to calculate the density and compared.

The population estimate of wildlife species requires the total area of potential habitat for extrapolating the density into population size. Defining the total potential habitat, based on the entire total of forest cover, is commonly used in the population estimate for gibbon species. However, a previous study suggested that not all forested areas are suitable for gibbons (Widyastuti et al. 2020). Some environmental variables limit the distribution of these small apes, such as elevation, vegetation characteristics, climatic factors and anthropogenic disturbances (Widyastuti et al. 2020). In this study, a habitat suitability model, which takes into account several environmental variables, was used to calculate the total area of potential habitat for Javan gibbons in Dieng. Furthermore, as gibbon densities could also be influenced by various environmental variables (Marshall 2009, Hamard et al. 2010, Cheyne et al. 2016, Hankinson et al. 2021), this study also tested differences in Javan gibbon densities between the habitat suitability types and estimated the total population size separately according to habitat suitability type.

Identification of the specific environmental variables that influence gibbon density is important for defining strategies in Javan gibbon habitat management. Previous studies have analysed the relationship between vegetation characteristics and some other gibbon species. Most studies have revealed that the densities of other gibbon species correlated with canopy cover, tree height, the density of large trees (based on diameter at breast height [DBH]) and food availability (Hamard et al. 2010, Cheyne et al. 2016, Hankinson et al. 2021), whereas one study found no correlation between them (Ray et al. 2015). The present study also investigated the correlation between habitat characteristics (including vegetation structure and forest elevation) and Javan gibbon density in Dieng.

## Material and methods

### Study area

The Dieng Mountains are located in Central Java Province, Indonesia (109°32'–109°56'E and 7°04'–7°13'S). The study was conducted in the remaining 90–175 km^2^ of natural forest, which mostly covers the northern part of the mountains (Nijman and Van Balen 1998, Setiawan et al. 2012). The altitudinal range of 250–1700 m above sea level is covered by forest blocks from lowland to the submontane tropical rainforest. The area has a hilly terrain and complex riverine. The forested area consists of a mixture of natural and plantation forests dissected by a large number of secondary roads and most relatively flat areas amongst the forest patches were built as settlements and croplands. Most natural forest patches are secondary forests due to logging in the past and current non-timber extractions by local people (shade-grown coffee planting, honey collecting, firewood collecting etc.) (Setiawan et al. 2012). However, the other forest patches are topographically inaccessible. Although diverse flora and fauna inhabit the forest, especially for all primates in Java (Nijman and Van Balen 1998), this area received less protection for biodiversity conservation. All forested areas are administratively managed by Perum Perhutani and Perusahaan Perkebunan Negara (an Indonesian state-owned forestry and plantation enterprise), which are mainly responsible for forest production. Some forest blocks are protection forest, which are not converted to plantations to maintain soil fertility and prevent landslides (Nijman and Van Balen 1998). Plantation forests such as pine, teak, agathis, rubber and tea plantations are generated in the surrounding the area, mostly adjacent to the natural forest. Moreover, many natural features, such as rivers and waterfalls, have been developed as tourism parks.

### Defining the potential habitat using the habitat suitability model

This study used ecological niche modelling (Franklin 2010) to estimate the total habitat potentially occupied by Javan gibbons in the Dieng Mountains. The maximum entropy algorithm (Phillips et al. 2004, Phillips and Dudık 2008) was generated for the modelling in the MaxEnt 3.4.4 programme (Phillips et al. 2020). The model has been generated in a previous study (Widyastuti et al. 2020), but it was re-run in the present study with improved environmental variables. The total of nine independent environmental variables were involved as predictors (Table 1). The model used the 70% (214) of recorded occurrence points during the field survey in 2018 as a training sample (see Widyastuti et al. 2020). The model was run under 10 replicates, a subsample replicated run type and used the 10 percentile training presence as a threshold rule (Young et al. 2011). The value from area under the curve (AUC) of the receiver operating characteristic (ROC) was used to evaluate the model performance (Peterson et al. 2011).

The index produced from the model ranged from 0 to 1, with 1 indicating the highest probability of Javan gibbon presence. The map with the probability range was then delineated into suitable and unsuitable habitats, based on the 10% training presence logistic threshold (Young et al. 2011, Fitzgerald et al. 2018), which is 0.27. The suitable habitat was expected to be the area where Javan gibbons potentially occur. The suitable habitat was then divided equally into two types, low suitable and high suitable. The 91 remaining occurrence points were then used for validation.

### Study design

**Figure 1.**
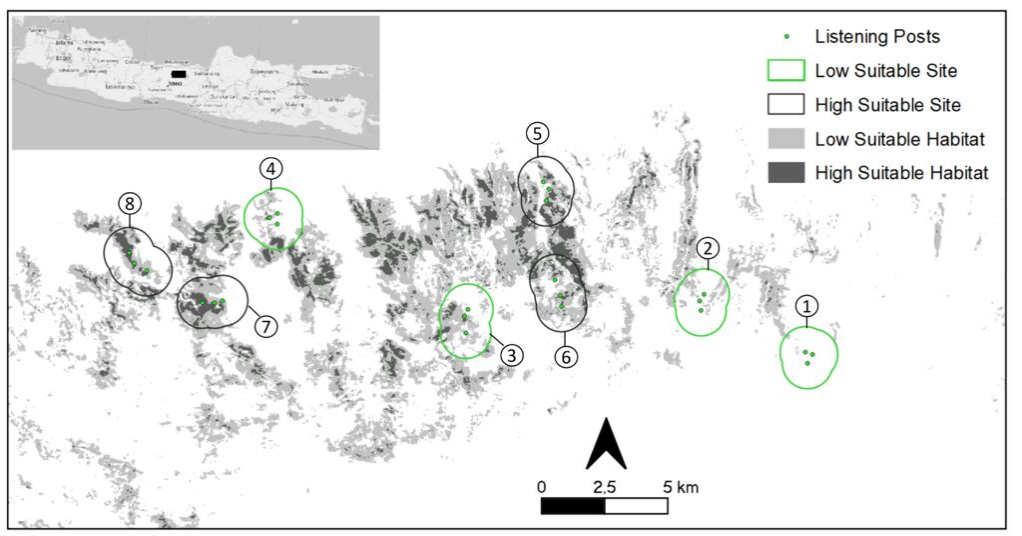
Study area and sampling sites (1 km buffer from each listening post) within the Javan gibbon habitat suitability type in the Dieng Mountains, Central Java, Indonesia. (Sampling sites: 1: Sikesod; 2: Tombo; 3: Sawangan Ronggo; 4: Sawahan; 5: Salakan; 6: Tinalum; 7: Kalipaingan; 8: Linggo Asri).

The fixed-point count was used owing to its efficiency and suitability for surveying gibbons, based on its call (Brockelman and Srikosamatara 1993, O'Brien et al. 2004, Whittaker 2005, Buckley et al. 2006, Cheyne et al. 2008, Cheyne et al. 2016, Hamard et al. 2010, Kabir et al. 2021). At each sampling site, three listening posts (LPs) were located on an either linear or triangular formation on the ridge or at a higher elevation to cover a wider listening area free from disturbing noise (Brockelman and Ali 1987, Cheyne et al. 2008, Höing et al. 2013). The posts were positioned 300–500 m apart to maximise the listening area and to allow detection of groups from at least two LPs (Brockelman and Ali 1987, Kidney et al. 2016, Hankinson et al. 2021). The outmost LPs of the adjacent sampling sites were positioned ≥ 2 km away from each other to avoid counting the same gibbon group at more than one site. This study used a 1-km buffer around each LP as effective listening radius (ELR) to define the effective listening area (ELA) of each sampling site (Brockelman and Ali 1987, Cheyne et al. 2008, Hamard et al. 2010) (Fig. 1). According to the identified habitat suitability type (Table 2), four sampling sites were placed in the area where the percentage of high suitable habitat was relatively high (≥ 19%), namely high suitable sites and four sampling sites were placed in the area where the percentage of high suitable habitat was relatively low (≤ 6%), namely low suitable sites (Fig. 1).

### Auditory sampling

The auditory sampling in each site was sequentially conducted from 5 October to 20 November 2021. Two observers sat at each LP in one sampling site to record the gibbon calls simultaneously. At each post in each site, each gibbon call heard between 06:00–10:00 h for four consecutive days were recorded, including the compass bearing, start and end times and number of female great calls (Brockelman and Srikosamatara 1993, Cheyne et al. 2016). The data collection was started at 06:00 h, when female gibbons in Dieng most frequently call (Geissmann and Nijman 2001). Data were not collected during rainy or stormy days and when the rain had continued for 2 h before the start of sampling, as rain and strong winds can affect negatively gibbon vocalisation (Brockelman and Srikosamatara 1993, Cheyne et al. 2007).

### Data mapping and group identification

Each gibbon call recorded per day, based on compass bearing, was plotted into the map using ET GeoWizard in ArcMap 10.5. Imaginary lines represented the compass bearing of each call recorded. The lines were then imported to Google Earth Pro for group identification. Only calls that consist the female great call (indicating a gibbon group) were included in the analyses to avoid counting solitary gibbons. First, the bearing lines from two or more LPs were paired when the singing timeframe and number of great calls were closely similar. If the paired lines produced an intersection or triangulation, the intersected point was then identified as the estimated singing location. Finally, gibbons at two or more singing locations were identified as the same group if the locations were within 500 m of each other. Otherwise, they were identified as different groups if they sang at the same time or too close in time to be from the same group (Brockelman 2019, Brockelman and Ali 1987, Brockelman and Srikosamatara 1993, Cheyne et al. 2016, Hankinson et al. 2021, O'Brien et al. 2004) or if confirmed with other evidence (e.g. identification by sighting or fragmented by an unforested barrier).

### Density data calculations

All recorded Javan gibbon calls, including non-triangulated calls, which follow these conditions were used in the group density calculation: 1) consisted of great calls (female great call) as a representative of the family group, 2) had a compass bearing to the identified singing group location and estimated to be that particular group and 3) within an ELA (Nijman and Van Balen 1998, Geissmann and Nijman 2001). The gibbon group density data were calculated per array using the standard triangulation calculation (Brockelman and Ali 1987, Nijman 2004, Cheyne et al. 2008, Hamard et al. 2010) and an ASCR programme (Stevenson et al. 2021). However, on the basis of cases reported in previous studies (Hankinson et al. 2022), we used two alternative calculation methods for the ASCR calculation, which are explained in the Acoustic spatial capture-recapture subsection . As a result, this study compared the gibbon group densities obtained from three methods using paired *t* tests to analyse the differences between the methods (Hankinson et al. 2021).

#### Standard triangulation

Triangulation calculations were generated using the package developed by Vu and Rawson (2011) in Excel Spreadsheet (Microsoft Office Professional 2013). This calculation package is based on the standard triangulation formula (Brockelman and Ali 1987): \begin{varwidth}{50in}
        \begin{equation*}
            D= \frac{n}{[p(m){\times}ELA]}
        \end{equation*}
    \end{varwidth}, where *D* is the gibbon group density at an array in group/km^2^, *n* is the number of groups heard within the listening area (*E*) at a particular array during the sampling period (4 days) and *p(m)* is the calling probability as a correction factor at an array over the sampling period of days. A correction factor is needed for this method because of the possibility of a group that made no calls and then were not detected during the sampling period (Brockelman and Ali 1987, Brockelman and Srikosamatara 1993). The correction factor *p(m)* was calculated for each site using the following formula: \begin{varwidth}{50in}
        \begin{equation*}
            p(m)=1-[1-p(1)]^m
        \end{equation*}
    \end{varwidth}, where *p(1)* is the calling or detection probability for any given day and *m* is the number of survey days. *p(1)* was calculated from the survey data in each array, based on the 3-day survey in accordance with the calculation protocol (Vu and Rawson 2011). The ELA was calculated for each array by creating a buffer with a fixed ELR of 1 km around each LP and then three combined buffer zones were calculated as ELA. Areas not covered by forest were excluded from ELA (Hamard et al. 2010, Hankinson et al. 2021).

#### Acoustic spatial capture-recapture

The gibbon group density per array was also estimated using the ASCR package (Stevenson et al. 2021) in an online interface application (Jones-Todd 2022). The mapped calls within ELA, as loaded in the triangulation calculation, were set into the daily detection data, which listed only one detection for each group at each post on each day. The detection data consisted of the occasion or survey day, post identity and group identity. Bearing data were also included to increase the precision of the density estimate (Stevenson et al. 2021). The models per array were run separately using a 1000-m mask, 15-m spacing and half-normal distribution and by fixing the g0 (probability of detection at 0 m from the post) at 1 (Hankinson et al. 2021).

The *D* estimated from the ASCR programme should be divided by the number of survey days and then by the daily calling probability *p(1)* to obtain the group density in a site (Hankinson et al. 2022, Jones-Todd 2022). However, these steps were not applied for calculating the densities of *Hylobateslar* and *Symphalangussyndactylus* in North Sumatra in a previous study (Hankinson et al. 2021). This decision was made considering that the density before the application of the steps was more realistic than that after the steps were applied. It corresponded to the recorded numbers of calls and identified gibbon groups in each sampling site from the field (personal communication, Hankinson 2022). Therefore, we included two different ASCR approaches, before and after division and then compared the results.

### Group size and population size calculations

In the field, visual encounters were also recorded to estimate the group size. The number of individuals seen, age class, time and coordinates were recorded. Considering that the number of groups encountered in 2021 was not robust, the group size used for estimating the population size was calculated from the visual encounters in the survey in August–December 2018, which was conducted in a longer period and obtained a larger sample (Table 4). In addition, several groups monitored in 2019 and 2020 were also summarised to compare the group size data during the period 2018–2021.

Individual densities were calculated per site by multiplying the group density with the overall group size. Individual densities were averaged for each habitat type. The population size in each habitat type was calculated by multiplying the average individual density in each habitat type by the total area for the corresponding habitat type. The population sizes in the low and high suitable habitats were then summed to obtain the overall population size.

### Habitat characteristics and correlation with gibbon density

Habitat characteristics were measured in 10 plots of 10 x 10-m vegetation plots randomly placed within the ELA of the eight sampling sites (Hamard et al. 2010, Hankinson et al. 2021). The data on the plots were measured at the same period with the population survey. The following variables for the trees with a DBH of ≥ 10 cm were recorded: 1) DBH (cm), the perimeter measured using a measuring tape and then converted to diameter; 2) tree height (m), measured using a Nikon Laser Rangefinder; 3) crown area (m^2^), calculated using following formula: \begin{varwidth}{50in}
        \begin{equation*}
            A=π(\frac{N-S width}{2})x(\frac{E-W width}{2})
        \end{equation*}
    \end{varwidth}, 4) canopy cover (%), measured at each corner and in the middle of the plot using visual estimation by the same observer for all plots; and 5) tree density (number of trees per km^2^) (Hamard et al. 2010, Kim et al. 2011, Hankinson et al. 2021). The elevation for each plot was also recorded from the GPS receiver.

All habitat characteristics and group density data were tested for normality using the Shapiro-Wilktest. After the normality was confirmed for all data, the differences in habitat characteristics between the sites were evaluated using one-way analysis of variance (ANOVA) test. The correlation between the gibbon group density and each habitat characteristic was examined using the Pearson correlation test. All statistical analyses were generated in R studio version 2022.02.3 then delineated into suitable and unsuitable habitats, based on the 10% training presence logistic threshold (Young et al. 2011, Fitzgerald et al. 2018), which is 0.27. The suitable habitat was expected to be the area where Javan gibbons potentially occur. The suitable habitat was then divided equally into two types, low suitable and high suitable. The 91 remaining occurrence points were then used for validation.

## Results

### Suitable habitat and habitat types

The Maxent model produced the probability index which ranged from 0 to 0.96. The range of the index was then reclassified into three habitat types, which were unsuitable, low suitable and high suitable habitats (Table 2). The model revealed that the overall potential habitat (total of low suitable and high suitable habitats) for Javan gibbons in the Dieng Mountains was 100.91 km^2^, where the low suitable habitat was larger than the high suitable habitat (Table 2). The Maxent model showed high model performance, based on an AUC value of 0.946 ± 0.007. Of the testing points, 90% were in the suitable habitat, mostly in the high suitable habitat (51.65%), which indicated the appropriate model classification.

### Density estimate

The total survey effort covered 33.4 km^2^ of Javan gibbon habitat (on the basis of the ELR of 1 km from each LP) across eight sites during the 32-day survey. A total of 529 call events were recorded from 24 LPs and 52 groups of Javan gibbons within all ELAs were identified. The number of groups identified in each site ranged from 2 to 10. The detection probability of Javan gibbons [*p(1)*, the probability of calls produced on any given day] during the 3-day survey in each array, ranged from 0.38 to 0.74. The correction factors [*p(m)*, the proportion of gibbons expected to sing at an area during the sampling period] for the 4-day survey in each site ranged from 0.85 to 0.99.

The group densities for each sampling site that were calculated by triangulation, ASCR before division and ASCR after division were within the ranges of 1.68–7.45, 0.7–5.9 and 0.46–2.68 (in groups/km^2^), respectively (Table 3), which showed normal distributions (Shapiro-Wilk test: *P* = 0.5358, *P* = 0.1867 and *P* = 0.6106, respectively). The group density calculated by triangulation and ASCR before division were not significantly different (paired *t* test: *t* = 0.85, *df* = 7, *P* = 0.422). By contrast, the density calculated by ASCR after division was significantly lower than those calculated using the other calculation methods (paired *t* test with triangulation: *t* = 5.34, *df* = 7, *P* = 0.00107; paired *t* test with ASCR before division: *t* = 4.86, *df* = 7, *P* = 0.00183). The mean density in each habitat type (between low and high suitable sites) were significantly different for all calculation methods (ANOVA test: *P* = 0.0195 for triangulation, *P* = 0.00297 for ASCR before division and *P* = 0.00604 for ASCR after division). Considering that the traditional triangulation method is the most common method to calculate gibbon density and the ASCR is a newly-developed statistical programme, the group densities calculated using ASCR before division were used for further analyses. The mean density was 2.15 groups/km^2^ in the low suitable habitat and 5.55 groups/km^2^ in the high suitable habitat (Table 3).

### Group size and population estimates

During the fieldwork in 2021, more than nine groups were encountered, but only nine groups from five sites could be observed and counted accurately. However, during the previous work in 2018, more extensive encounters led to the identification and confirmation of 20 groups from nine sites. Finally, we used the group size from 2018 to calculate the population size in this study, considering the larger sample size and coverage of the sampling sites. The number of individuals per group ranged from 2–7 and the mean group size was 3.95 individuals per group. A number of infants and juveniles were observed during the period 2018–2021 (Table 4).

The total suitable habitat for Javan gibbons was calculated to be 100.91 km^2^, of which 83.41 km^2^ was low suitable habitat and 17.50 km^2^ was high suitable habitat. The number of Javan gibbons was estimated to be 708 in the low suitable habitat, 384 in the high suitable habitat and 1092 in the whole area of the Dieng Mountains (Table 5).

### Correlation density with habitat characteristics

All data on the habitat characteristics followed a normal distribution (Table 6). Significant differences amongst sites were found only for elevation (ANOVA test: *P* = 0.04), whereas all vegetation characteristics did not significantly differ amongst sites (Table 6). No significant correlations were found between density and all habitat characteristics tested in this study (Table 7).

## Discussion

### Density and population size

Although the Dieng Mountains are a heterogenous and unprotected landscape, these areas hold a large portion of the Javan gibbon population (Nijman 2004). After a decade since the last study that updated the population size, this study found a significantly higher density, group size and population size of Javan gibbons in the Dieng Mountains than any previous studies (Table 8). The mean density in the low suitable site (2.15 groups/km^2^) demonstrates an ideal range of gibbon density (2–5 groups/km^2^) (Brockelman and Srikosamatara 1993) and is still within the same range as those reported in previous studies. However, the mean density in the high suitable sites (5.55 groups/km^2^) was the highest amongst those from any surveys in Dieng and demonstrates a high density range (5–6 groups/km^2^) (Brockelman and Srikosamatara 1993) (Table 8).

The higher Javan gibbon density in the present study than in the most recent study indicates two possibilities. First, it indicates that the Javan gibbon population in Dieng has been increasing. Alternatively, the population has not been increasing, but the higher density in the recent study resulted from the difference in survey technique. Compared with the most recent study (Setiawan et al. 2012), which used the visual line-transect method, this study used a fixed-point count method, based on auditory sampling. As Javan gibbons are fully arboreal and fast, but smoothly moving primates, they can flee before being detected under visual observation inside the dense forest; thus, some gibbons might have been missed in the count in the previous study (Brockelman 2019). Our survey using auditory sampling could probably detect more gibbon groups than the previous survey, but we cannot conclude that the previous study underestimated the number of groups on the basis of this reason alone. Regarding the difference in survey method, overestimation was more unlikely in the recent study for two reasons. First, we carefully used a conservative principle to count the number of gibbon groups within the sampling area. We only separated two or more singing locations as different groups if they met the set criteria. Second, we heard and recorded high numbers of gibbon calls in the sampling sites that showed very high group densities, such as Salakan and Kalipaingan and recorded very low numbers of calls in the sites that showed very low densities, such as Sikesod, for which 0.7 group/km^2^ was recorded (Table 3).

Without any other robust supportive evidence, we cannot conclude that the Javan gibbon density has not been increasing. On the other hand, this study shows a significantly higher group size than any other previous study of Javan gibbons in Dieng (Geissmann and Nijman 2001, Nijman 2004, Setiawan et al. 2012), which was 3.95 individuals per group (n = 20 groups) in 2018 (Table 4). In all studies, the group size was commonly determined from visual encounter; therefore, it is the best parameter for comparison between studies. The group size in the present study is an increase from that in the previous study (Setiawan et al. 2012), which reported that the mean group size in several sites in Dieng was only 2.61 individuals (n = 31 groups). An earlier study (Geissmann and Nijman 2001) also reported a lower group size from the forest near Linggo Asri in Dieng, which was 3.5 individuals (n = 15). In addition, regeneration was observed. A total of nine groups were found with offspring in 2018, of which seven had juveniles, one had an infant and one had both a juvenile and an infant. Several numbers of offspring were also found in several groups encountered in 2019–2021 (Table 4). Thus, this indicates that the first possibility is more likely. The Javan gibbon population in Dieng has been increasing or has remained at least stable during the last decade.

The population increase suggests that the reproduction rate of Javan gibbons in the Dieng Mountains during the last decade was higher than their mortality rate. The larger group size and number of offspring found in this survey prove the high reproduction rate. Healthy reproduction is supported by enough food sources. Therefore, the high reproduction rate indicates that the food tree species for Javan gibbons in the Dieng Mountains were plentiful, although more work is needed to confirm this. Furthermore, the availability of habitat space for the territory of new groups is also important for the population growth of Javan gibbons. On the basis of the Javan gibbon home range size of 12–36 ha (Iskandar 2007, Malone 2007, Kim et al. 2011, Maya 2013), the identified suitable habitat of 100 km^2^ (equal to 10,000 ha) could roughly support 400 gibbon groups (with the assumption of a 25-ha home range size), but the estimate in this study was only 276 groups. This means that the Dieng Mountains have provided enough habitat space for new gibbon groups in the last decade and can still support the population growth in the future.

The Dieng Mountains are not protected under a conservation area and consist of heterogenous land-use and land-cover. This situation has led to various levels of disturbance that threaten gibbons and their habitats. However, this study suggests that the rate of mortality or disappearance due to illegal hunting of Javan gibbons in the Dieng Mountains has been lower than their natality rate in the last decade. This might be a result of the wide range of conservation efforts to reduce the threats at the grassroots level by various parties, mostly by a local non-government organisation (NGO; SwaraOwa), during the last decade. Several long-term conservation activities have been implemented, including livelihood development, environmental education, community awareness and ecological research (Setiawan et al. 2009, Herdiansyah and Setiyono 2019, Setiawan et al. 2020). In particular areas, such as near the Sokokembang forest, there is an indication that the local people have become more aware of the need to protect these endangered primates from illegal hunting. Furthermore, no case of Javan gibbon illegal hunting near Sokokembang has been reported during the last decade (Hendriati et al. 2020).

### New approaches

The IUCN Species Survival Commission Primate Specialist Group's Section on Small Apes recommended the recently-developed ASCR for gibbon population studies, as it is the most accurate way to analyse acoustic data (Cheyne, unpublished data; Hankinson et al. 2021). However, standard triangulation was still used in this study to calculate the gibbon group density because it has an important basic concept in density estimation using vocal count data (Brockelman and Ali 1987, Hankinson et al. 2021) and has been proven to be reliable in several surveys (Brockelman and Srikosamatara 1993, Nijman 2004, Cheyne et al. 2008, Höing et al. 2013, Gilhooly et al. 2015). Our results show that dividing the *D* from ASCR by the number of survey days, which was four and by the probability of gibbon calls in a day [*p(1)*] resulted in a significantly lower density than the original *D* before the divisions. Moreover, the densities obtained before the divisions were most likely to represent the number of calls heard and the groups identified during the survey. Furthermore, the densities obtained using ASCR before the divisions were within the same range as the densities obtained using the basic method, standard triangulation. For this reason, we used the densities from the ASCR before the divisions for the population size calculation and correlation analyses with habitat characteristics. The finding that the *D* of ASCR before division is more realistic than that of ASCR after division was similar to the findings of previous studies for lar gibbon (*Hylobateslar*) and siamang (*Symphalangussyndactylus)* in north Sumatra (Hankinson et al. 2021; personal communication, Hankinson 2022), but not for Thomas' langurs (Presbytis thomassi) (Hankinson et al. 2022). By contrast, in accordance with the instruction on the manual web-page of ASCR to divide the *D* by the number of survey days and *p(1)*, the langur group densities appeared to be realistic and comparable with the results of a previous studies (Hankinson et al. 2022). This study indicates that the ASCR method is a useful modelling tool for calculating gibbon or primate density, based on calls, although the steps to divide the *D* by the number of survey days and *p(1)* may give different results in different species at different study sites. Therefore, in order to calculate the gibbon group density, we suggest to carefully consider the *D* obtained using the ASCR method, both before and after division, by comparing it with other *Ds* that were obtained using the other calculation method, such as the standard triangulation or with other *Ds* from previous studies.

The density was converted into population size using two separate extrapolations, based on the habitat suitability model for the low and high suitable habitats. This approach revealed that the gibbon densities in the two habitat types were significantly different. Thus, extrapolating by averaging the densities from all sites will result in bias estimation. The previous Javan gibbon population studies used separate extrapolations, based on altitudinal range (e.g.,] Kappeler (1984), Asquith et al. (1995), Nijman and Van Balen (1998)). However, the latest studies also support that elevation was one of the most important predictors of the presence of Javan gibbons, but there were other environmental variables which were also important, such as anthropogenic disturbances (Widyastuti et al. 2020). In the current condition in Dieng, not all lowland forests, which provide better support for higher gibbon densities than high-elevation forests, have good-quality vegetation and are free from anthropogenic disturbances. In our study, the group density in Sawahan, which is lowland forest, was much lower than those in other lowland sites. Therefore, the combination of multiple variables, rather than elevation alone, was taken into account in the extrapolation.

### Gibbon density and habitat characteristics

Our study did not find any correlation between the gibbon density in the Dieng Mountains and any of the habitat characteristics tested (Table 7). Although the density significantly differed between the habitat suitability types, it was not influenced by the vegetation structure or forest elevation. All vegetation characteristics did not show significant differences between the sites (Table 6). This indicates that the forest structures throughout the Dieng Mountains were similar and had no effect on the density variation. The same pattern of vegetation structure and no effect on gibbon density were also found in another study on western hoolock gibbons (*Hoolockhoolock*) in Namdapha National Park, India (Ray et al. 2015).

Other vegetation characteristics such the availability of food trees might have more significant influences on gibbon density. However, we could not examine this because of the lack of Javan gibbon food data and limitation in tree species identification. The tree species were identified with non-standardised local names by the experienced local guide; thus, the names given could differ between the sites. Therefore, the study of Javan gibbon dietary ecology that includes an analysis of vegetation composition in the Dieng Mountains is crucial for further research. Alternatively, as our study used multiple variables in the habitat type classification, anthropogenic disturbance or climatic factors could also be the major causes of the gibbon density variation. Statistical analyses that take into account multiple variable combinations to investigate the major causes of the density variation are recommended for further study.

### Implication for conservation

This study found high gibbon group densities and estimated a large number of Javan gibbons inhabiting the Dieng Mountains. However, threats to Javan gibbons remain, the risk of population decline is still high and even local extinction is still possible. Long-term population monitoring should be strengthened along with other conservation programmes to ensure the long-term future of this endemic species. Long-term population monitoring is important to monitor the trend of the population size and also to detect the probability of local extinction in the early stage, which could be caused by hunting or deforestation (Vu et al. 2020).

Although our study indicates that the conservation efforts during last decade have led to the increase the Javan gibbon population in Dieng, more efforts are crucial to ensure the long-term future of this local population. The habitat degradation in some locations has been and will be a potential problem for Javan gibbons. For example, the forest surrounding Sawahan has been degraded because of the priority for unsustainable agroforestry, which has resulted in a low gibbon density. Furthermore, some forest patches in Kalipaingan and Linggo Asri hold high gibbon densities, as they are topographically inaccessible, but the remaining areas are prioritised for unsustainable agriculture. Conservation programmes, such as environmental education and community development for the villages surrounding these locations, should be strengthened to raise awareness and shift livelihoods to more sustainable ones. In addition, a new threat has arisen. In the past 5 years, the local people surrounding this Javan gibbon habitat, supported by the local government, developed many natural attractions which successfully attracted massive numbers of tourists. However, although this provides an alternative strategy to improve the local economy for the benefit of the local people, this poses a serious threat to Javan gibbons and their habitat, unless wise and careful management is employed. Gibbon watching as a nature-based tourism is a good tourism programme, which has been initiated in Petungkriyono (Supriatna et al. 2022). This provides an alternative sustainable tourism to this district and could be combined with sustainable or wildlife-friendly products of forest commodities to improve the local economy.

The results of this study strongly support that the Dieng Mountains are an important habitat for Javan gibbons and hold a significant proportion of the total population of gibbons in Java. Since the previous study, the forest in the Dieng Mountains has been suggested to be designated as a protected area (Nijman and Van Balen 1998), but this appears not to be a good solution for such a heterogenous landscape and complicated condition, as explained earlier. Many people live adjacent to the forest, who need to fulfil their economic well-being. Furthermore, a win-win solution should be attempted to allow the regulation of both interests, which are conserving the biodiversity in the Dieng Mountains, including Javan gibbons and improving the local economy. Since 2018, the local NGO (SwaraOwa) and local government have proposed a 5173.80 ha forest area in Petungkriyono for a collaborative forest management called *Kawasan Ekosistem Esensial*, or essential ecosystem area (EEA), to protect the habitat of Javan gibbons and the biodiversity in the Dieng Mountains. The EEA can be managed collaboratively by a forum of various stakeholders mainly to protect the biodiversity and ecosystem in the area and to manage the ecosystem sustainably for economic development (Sahide et al. 2020, Rosdiana et al. 2022). Although it only involves a small portion of the natural forest in the Dieng Mountains, it is a good decision to start with and can be expanded to other forest patches in the future.

## Conclusions

This study estimated that the Javan gibbon population in the Dieng Mountains has most likely increased. The mean group density in each habitat type was high. The gibbon density was estimated to be 2.15 groups/km^2^ in the low suitable habitat and 5.55 groups/km^2^ in the high suitable habitat. The mean group size reported in this study (3.95, n = 20) was higher than those in previous studies. The overall population size was estimated to be 1092 gibbons, with 708 gibbons in the low suitable habitat and 384 in the high suitable habitat. This study did not find any relationship between Javan gibbon density and habitat characteristics, including vegetation characteristics and forest elevation.

## Figures and Tables

**Table 1.** Environmental variables as predictors in the MaxEnt ecological niche modelling for the Javan gibbons in the Dieng Mountains.

No	Category	Variables	Data Source and Spatial Resolution	Acquisition Date
1	Climate	Land surface temperature	Landsat 8 images (*thermal band*), 30 m	Median 2017–2018
2	Topography	Elevation	DEMNAS *digital elevation model*, 8.33 m	2019
3		Slope	DEMNAS *digital elevation model*, 8.33 m	2019
4	Vegetation	Normalised difference vegetation index (NDVI)	Sentinel–2 images, 10 m	August–December 2018
5		Natural forest cover	Landsat 8 images, 30 m	Median 2018
6	Disturbance	Distance to crop	Landsat 8 images, 30 m	Median 2018
7		Distance to plantation	Landsat 8 images, 30 m	Median 2018
8		Distance to settlement	Landsat 8 images, 30 m	Median 2018
9		Distance to road	Rupa Bumi Indonesia	2019

**Table 2.** Classification of suitability type of the Javan gibbon habitat in the Dieng Mountains.

Habitat Suitability Type	Index Range	Validation Occurrence Point (%)	Total Area (km^2^)
Unsuitable	0–0.28	9.89	
Low Suitable	0.28–0.62	38.46	83.41
High Suitable	0.62–0.96	51.65	17.50

**Table 3.** Javan gibbon group density, based on the standard triangulation and acoustic spatial capture-recapture (ASCR) methods in the Dieng Mountains, 2021. ELA: Effective listening area with a fixed radius of 1 km. CI: Confidence Interval. ^A^Divided by the number of survey days (4) and by *p(1)*. *Significantly different at the 0.05 level. **Significantly different at the 0.01 level.

Habitat Type	Sampling Site	Groups Heard (n)	*p(1)*	*p(m)*	ELA (km^2^)	Density Triangulation (groups/km^2^)	Density ASCR (groups/km^2^) (2.5% Cl) (97.5% Cl) Before^A^	Density ASCR (groups/km^2^) After^A^	AIC Value ASCR
Low Suitable Sites	1 Sikesod	2	0.38	0.85	4.18	1.68	0.7 (−0.09 to 1.5)	0.46	30.1
	2 Tombo	3	0.48	0.92	4.38	1.73	2.1 (0.4–3.7)	1.1	84.8
	3 Sawangan R.	7	0.7	0.99	4.66	4.11	3.9 (2.1–5.7)	1.39	147.1
	4 Sawahan	4	0.52	0.95	3.84	3.03	1.9 (0.6–3.2)	0.92	78.1
	Average					2.64*	2.15**	0.97**	
High Suitable Sites	5 Salakan	10	0.53	0.95	3.85	6.55	5.7 (3.3–8.2)	2.68	199.9
	6 Tinalum	8	0.69	0.99	4.76	4.24	5.9 (2.7–9.0)	2.15	213.1
	7 Kalipaingan	10	0.54	0.96	4.21	7.45	5.8 (3.5–8.0)	2.67	254.5
	8 Linggo Asri	8	0.74	0.99	3.52	4.56	4.8 (2.7–6.9)	1.63	171.9
	Average					5.70*	5.55**	2.28**	
	Total	52			33.4				

**Table 4.** Javan gibbon group size and minimum number of offspring from visual encounters between 2018 and 2021.

Survey Year	Number of Groups	Mean Group Size	Juvenile	Infant	Observer
2018	20	3.95	8	2	SW and AS
2019	9	3.30	0	2	AS
2020	8	2.88	3	1	NA
2021	9	3.78	3	1	SW and NA
Average		3.48			

**Table 5.** Population estimates of Javan gibbons in the Dieng Mountains (based on the density calculated using ASCR before division).

Habitat Type	Group Density (groups/km^2^)	Population Density (Individuals/km^2^)	Total Area of Habitat Type (km^2^)	Number of Groups	Population Size
Low Suitable	2.15 (0.7–3.9)	8.49 (2.8–15.4)	83.41	179	708 (231–1284)
High Suitable	5.55 (4.8–5.9)	21.92 (18.9–23.3)	17.50	97	384 (331–407)
**Total**			**100. 91**	**276**	**1092 (563**–**1692)**

**Table 6.** Differences in habitat characteristics between sites. *Significantly different at the 0.05 level.

Site	DBH (cm)	Tree Height (m)	Crown Area (m^2^)	Canopy Cover (%)	Tree Density (number of trees/km^2^)	Elevation (m a.s.l.)
Sikesod	27.85	14.54	22.11	64.50	87000	1376
Tombo	26.63	13.11	18.34	65.50	89000	1281
Sawangan Ronggo	42.07	13.79	43.83	59.40	56000	899
Sawahan	43.98	13.32	36.34	58.25	55000	516
Salakan	42.32	14.42	13.26	81.65	57000	485
Tinalum	59.44	18.69	40.13	64.25	52000	894
Kalipaingan	24.78	11.56	27.10	62.18	98000	744
Linggo Asri	31.90	12.36	37.55	68.25	85000	550
Shapiro-Wilk *P*	0.29	0.10	0.54	0.06	0.07	0.24
ANOVA *P*	0.68	0.82	0.46	0.65	0.94	0.04*

**Table 7.** Correlation between the habitat variables and the densities (calculated by ASCR before division).

Habitat Characteristic	Density Triangulation		Density ASCR	
	Pearson Correlation	*P*	Pearson Correlation	*P*
Elevation	−0.671	0.063	−0.582	0.130
DBH	0.071	0.868	0.361	0.379
Tree Height	−0.182	0.666	0.171	0.686
Tree Density	−0.020	0.962	−0.222	0.598
Crown Area	−0.034	0.936	0.156	0.713
Canopy Cover	0.375	0.359	0.389	0.341

**Table 8.** Comparative density and population size of Javan gibbons in the Dieng Mountains. ^a^ and ^b^ correspond to the authors in the Reference column. ^A^In a low suitable habitat. ^B^In a high suitable habitat. *Converted from individual density and group size.

Method	Survey Year	Group Size (n)	Group density (groups/km^-2^)	Individuals Density (number of ind./km^2^)	Potential Habitat (km^2^)	Population Size	Reference
Fixed Point Count	1994–1995	-	0.9–1.1	1–7^a^ 3.0–3.6^b^	120–135	519–577	^a^Nijman and Van Balen (1998); ^b^Nijman (2004)
Fixed Point Count	1998	3.50 (15)	1.9–3.7	6.7–13.1	-	-	Geissmann and Nijman (2001)
Line Transect	2009–2010	2.61 (31)	1.97*	5.2	166.90	881	Setiawan et al. (2012)
Fixed point count	2018 and 2021	3.95 (20)	2.15^A^ (0.7–3.9) 5.55^B^ (4.8–5.9)	8.49^A^ (2.8–15.4) 21.92^B^ (18.9–23.3)	100.91	1092 (563–1692)	Present Study

## References

[B8444731] Asquith N. M., Martarinza, Sinaga R. M. (1995). The Javan gibbon (*Hylobatesmoloch*): status and conservation recommendations. Tropical Biodiversity.

[B8444444] Brockelman Warren, Ali R, Mittermeier R. A., Walsh C. W. (1987). Primate conservation in the tropical rain forest.

[B8443780] Brockelman Warren Y., Srikosamatara Sompoad (1993). Estimation of density of gibbon groups by use of loud songs. American Journal of Primatology.

[B8444552] Brockelman Warren Y. (2019). Counting gibbons: The evolution of sample methods. Interdisciplinary Research Review.

[B8444457] Brockelman Warren Y, Srikosamatara Sompoad (1993). Estimation of density of gibbon groups by use of loud songs. American Journal of Primatology.

[B8444229] Buckley Cara, Nekaris K A I, Husson Simon John (2006). Survey of *Hylobatesagilisalbibarbis* in a logged peat-swamp forest : Sabangau catchment , Central Kalimantan. Primates.

[B8444405] Cheyne S. M., Chivers D. J., Sugardjito J. (2007). Covarvariation in the great calls of rehabilitant and wild gibbons (*Hylobatesalbibarbis*). The Raffles Bulletin of Zoology.

[B8444312] Cheyne S. M., Thompson C. J.H., Phillips Abigail C., Hill Robyn M. C., Limin Suwido H. (2008). Density and population estimate of gibbons (*Hylobatesalbibarbis*) in the Sabangau catchment, Central Kalimantan, Indonesia. Primates.

[B8444524] Cheyne S. M., Gilhooly Lauren J., Hamard Marie C., Höing Andrea, Houlihan Peter R., Kursani, Loken Brent, Phillips Abigail, Rayadin Yaya, Capilla Bernat Ripoll, Rowland Dominic, Sastramidjaja Wiwit Juwita, Spehar Stephanie, Thompson Claire J. H., Zrust Michal (2016). Population mapping of gibbons in Kalimantan, Indonesia: Correlates of gibbon density and vegetation across the species' range. Endangered Species Research.

[B8444218] Djanubudiman Guritno, Arisona Jarot, Setiadi M Iqbal, Wibisono Farid, Mulcahy Glen, Indrawan Mochamad, Hidayat R M (2004). Current distribution and conservation priorities for the Javan Gibbon (*Hylobates moloch*).

[B8444277] Fitzgerald Maegan, Coulson Robert, Lawing A. Michelle, Matsuzawa Tetsuro, Koops Kathelijne (2018). Modeling habitat suitability for chimpanzees (*Pantroglodytesverus*) in the Greater Nimba Landscape, Guinea, West Africa. Primates.

[B8445117] Franklin J (2010). Mapping species distributions: Spatial inference and prediction.

[B8444691] Geissmann Thomas, Nijman Vincent, Nijman V. (2001). Forest (and) primates: Conservation and ecology of the endemic primates of Java and Borneo.

[B8444337] Gilhooly Lauren J, Rayadin Yaya, Cheyne Susan M (2015). Acomparison of hylobatid survey methods using triangulation on Müller’s Gibbon (*Hylobatesmuelleri*) in Sungai Wain Protection Forest, East Kalimantan, Indonesia. International Journal of Primatology.

[B8444396] Hamard Marie, Cheyne Susan M, Nijman Vincent (2010). Vegetation correlates of gibbon density in the peat-swamp forest of the Sabangau catchment, Central Kalimantan, Indonesia. American Journal of Primatology.

[B8444322] Hankinson Emma L., Hill Ross A., Marsh Christopher D., Nowak Matt G., Abdullah Abdullah, Pasaribu Nursahara, Supriadi, Nijman Vincent, Cheyne Susan M., Korstjens Amanda H. (2021). Influences of forest structure on the density and habitat preference of two sympatric gibbons (*Symphalangussyndactylus* and *Hylobateslar*). International Journal of Primatology.

[B8444614] Hankinson Emma, Korstjens Amanda H., Hill Ross A., Wich Serge A., Slater Helen D., Abdullah Abdullah, Supradi Supradi, Marsh Christopher D., Nijman Vincent (2022). Effects of anthropogenic disturbance on group densities of Thomas' langurs (*Presbytisthomasi*) within a lowland tropical forest, North Sumatra. Ecological Research.

[B8444664] Hendriati I T, Abdoellah O S, Parikesit (2020). Poaching di hutan Petungkriyono : perspektif ekologi politik.

[B8444628] Herdiansyah Ikbal, Setiyono Budi (2019). Pemberdayaan dalam perspektif pembangunan berkelanjutan: Studi kasus strategi pemberdayaan masyarakat Hutan Sokokembang LSM SwaraOwa di Kabupaten Pekalongan. Journal of Politic and Government Studies.

[B8444414] Höing Andrea, Quinten Marcel C, Indrawati Yohana Maria, Cheyne Susan M, Waltert Matthias (2013). Line transect and triangulation surveys provide reliable estimates of the density of Kloss’ gibbons (*Hylobatesklossii*) on Siberut Island, Indonesia. International Journal of Primatologi.

[B8444388] Iskandar Entang (2007). Habitat dan Populasi Owa Jawa (*Hylobatesmoloch* Audebert, 1797) di Taman Nasional Gunung Halimun-Salak Jawa Barat.

[B8444895] Jones-Todd C. M. (2022). ASCR Online Interface Package. https://cmjt.shinyapps.io/ascr_shiny/.

[B8444432] Kabir M Tarik, Ahsan M Farid, Cheyne Susan M, Sah Shahrul Anuar Mohd, Lappan Susan, Bartlett Thad Q, Ruppert Nadine (2021). Population assessment of the endangered Western Hoolock Gibbon *Hoolockhoolock* Harlan, 1834 at Sheikh Jamal Inani National Park, Bangladesh, and conservation significance of this site for threatened wildlife species. Journal of Threatened Taxa.

[B8444561] Kappeler Markus, Preuschoft H., Chivers D., Brockelman W. Y., Creel N. (1984). The lesser apes: evolutionary and behavioural biology.

[B8444513] Kidney Darren, Rawson Benjamin M, Borchers David L, Stevenson Ben C, Marques Tiago A, Thomas Len (2016). An efficient acoustic density estimation method with human detectors applied to gibbons in Cambodia. PLoS ONE.

[B8444238] Kim Sanha, Lappan Susan, Choe Jae C. (2011). Diet and ranging behavior of the endangered Javan gibbon (*Hylobatesmoloch*) in a submontane tropical rainforest. American Journal of Primatology.

[B8444247] Malone Nicholas Martin (2007). The socioecology of the critically endangered Javan gibbon (*Hylobatesmoloch*): Assessing the Impact of Anthropogenic Disturbance on Primate Social Systems.

[B8444574] Marshall Andrew J. (2009). Are montane forests demographic sinks for bornean white-bearded gibbons hylobates albibarbis?. Biotropica.

[B8444911] Maya R. I. (2013). Penggunaan habitat owa Jawa (*Hylobatesmoloch* Audebert, 1798) di Bukit Sirondo, Hutan Sokokembang, Petungkriyono, Pekalongan, Jawa Tengah.

[B8444200] Nijman Vincent, Van Balen S. (1998). A faunal survey of the Dieng Mountains, Central Java, Indonesia: Distribution and conservation of endemic primate taxa. Oryx.

[B8444209] Nijman Vincent (2004). Conservation of the Javan gibbon Hylobatesmoloch: Population estimates, local extinctions, and conservation priorities. Raffles Bulletin of Zoology.

[B8444544] Nijman Vincent (2020). Hylobates moloch.

[B8444466] O'Brien Timothy, Kinnaird Margaret, Nurcahyo Anton, Iqbal Mohamed, Rusmanto Mohamed (2004). Abundance and distribution of sympatric gibbons in a threatened Sumatran rain forest. International Journal of Primatology.

[B8444287] Peterson Andrew Townsend, Soberón Jorge, Pearson Richard G, Anderson Robert P, Martínez-Meyer Enrique, Nakamura Miguel, Araújo Miguel Bastos, Levin S. A., Horn H. S. (2011). Monographs in Population Biology.

[B8444268] Phillips Steven J., Dudík Miroslav, Schapire Robert E. (2004). A maximum entropy approach to species distribution modeling.

[B8444303] Phillips Steven J, Dudık Miroslav (2008). Modeling of species distributions with Maxent: new extensions and a comprehensive evaluation. Ecography.

[B8444927] Phillips S. J., Dudik M., Schapire R. E. (2020). Maxent software for modeling species niches and distributions. http://biodiversityinformatics.amnh.org/open_source/maxent/.

[B8444485] Ray Parimal, Kumar Awadhesh, Devi Ashalata, Krishna Murali, Khan Mohammed, Brockelman Warren (2015). Habitat characteristics and their effects on the density of groups of Western Hoolock gibbon (*Hoolockhoolock*) in Namdapha National Park, Arunachal Pradesh, India. International Journal of Primatology.

[B8444255] Roos Christian, Boonratana Ramesh, Supriatna Jatna, Fellowes John R., Groves Colin P., Nash Stephen D., Rylands Anthony B., Mittermeier Russell A. (2014). An updated taxonomy and conservation status review of Asian primates. Asian Primates Journal.

[B8444605] Rosdiana Rosdiana, Fatimah Siti, Riani Ditasya Anisa (2022). Legal protection of the Balikpapan Bay Essential Ecosystem Area. Jurnal Lex Suprema.

[B8444594] Sahide Muhammad Alif K., Fisher Micah, Nasri Nasri, Dharmiasih Wiwik, Verheijen Bart, Maryudi Ahmad (2020). Anticipating a new conservation bureaucracy? Land and power in Indonesia’s Essential Ecosystem Area policy. Land Use Policy.

[B8444637] Setiawan A, Nugroho T S, Wibisono Y, Ikawati V, Tasuri (2009). Project report to Rufford Small Grant Foundation: Conservation of endangered primates in Central Java, Indonesia. https://ruffordorg.s3.amazonaws.com/media/project_reports/2-66.05.09%20Final%20Report.pdf.

[B8444190] Setiawan Arif, Nugroho Tejo Suryo, Wibisono Yohannes, Ikawati Vera, Sugardjito Jito (2012). Population density and distribution of Javan gibbon (*Hylobatesmoloch*) in Central Java, Indonesia. Biodiversitas.

[B8444646] Setiawan Arif, Mujianto Meardhy, Harjanto Sidiq, Abdurrahman (2020). Final Report 2020: Coffee and Primate Conservation Project. https://swaraowa.org/wp-content/uploads/2022/01/Final-Report-Swaraowa_Coffee-and-Primate-Conservation-Project-2020.pdf.

[B8444496] Stevenson Ben C., Dam‐Bates Paul, Young Callum K. Y., Measey John (2021). A spatial capture–recapture model to estimate call rate and population density from passive acoustic surveys. Methods in Ecology and Evolution.

[B8444985] Supriatna J., Ario A, Setiawan A, Gursky S. L., Supriatna J., Achorn A. (2022). Ecotourism and Indonesia's Primates.

[B8444583] Vu Thinh T., Hoa Anh Nguyen Q., Rawson Benjamin M., Tran Dung V., Nguyen Hoa T., Van Thinh N. (2020). Monitoring occurrence, extinction, and colonization probabilities for gibbon populations. American Journal of Primatology.

[B8444505] Vu Tien Thinh, Rawson Benjamin Miles (2011). Package for calculating gibbon population density from auditory surveys. https://www.gibbons.asia/wp-content/uploads/2018/08/Guidelines-for-calculating-gibbon-population-density.pdf.

[B8444476] Whittaker Danielle (2005). Short Communication New population estimates for the endemic Kloss's gibbon *Hylobatesklossii* on the Mentawai Islands, Indonesia. Oryx.

[B8445209] Widyastuti S., Perwitasari-Farajallah D., Prasetyo L. B., Iskandar E., Setiawan A. (2020). Maxent modelling of habitat suitability for the endangered javan gibbon (*Hylobatesmoloch*) in less-protected Dieng Mountains, Central Java.

[B8444424] Young Nick, Carter Lane, Evangelista Paul (2011). A MaxEnt Model v3.3.3e Tutorial (ArcGIS v10).

